# Treating Small Bowel Obstruction with a Manual Physical Therapy: A Prospective Efficacy Study

**DOI:** 10.1155/2016/7610387

**Published:** 2016-02-18

**Authors:** Amanda D. Rice, Kimberley Patterson, Evette D. Reed, Belinda F. Wurn, Bernhard Klingenberg, C. Richard King, Lawrence J. Wurn

**Affiliations:** ^1^Clear Passage Physical Therapy, 4421 NW 39th Avenue, Gainesville, FL 32606, USA; ^2^Department of Mathematics and Statistics, Williams College, Williamstown, MA 01267, USA

## Abstract

Small bowel obstructions (SBOs) caused by adhesions are a common, often life-threatening postsurgical complication with few treatment options available for patients. This study examines the efficacy of a manual physical therapy treatment regimen on the pain and quality of life of subjects with a history of bowel obstructions due to adhesions in a prospective, controlled survey based study. Changes in six domains of quality of life were measured via ratings reported before and after treatment using the validated Small Bowel Obstruction Questionnaire (SBO-Q). Improvements in the domains for pain (*p* = 0.0087), overall quality of life (*p* = 0.0016), and pain severity (*p* = 0.0006) were significant when average scores before treatment were compared with scores after treatment. The gastrointestinal symptoms (*p* = 0.0258) domain was marginally significant. There was no statistically significant improvement identified in the diet or medication domains in the SBO-Q for this population. Significant improvements in range of motion in the trunk (*p* ≤ 0.001), often limited by adhesions, were also observed for all measures. This study demonstrates in a small number of subjects that this manual physical therapy protocol is an effective treatment option for patients with adhesive small bowel obstructions as measured by subject reported symptoms and quality of life.

## 1. Introduction

Small bowel obstruction (SBO) is a common life-threatening complication of surgery or abdominal trauma, typically caused by adhesions that form as a normal part of the healing process. When healing from surgery, an inflammatory response is initiated to recruit the cells necessary to close the surgical incision and repair the tissues. As a side effect of this inflammatory response, adhesions form in tissues at and near the surgical repair due to the presence of collagen and scar tissue mediators. Adhesions have been suggested to begin forming within hours after abdominal surgery; complications related to surgery are common in both pediatric and adult populations [1–3].

Surgery is frequently cited as the primary cause of bowel obstruction [4]. In 2010, 381,364 patients in the United States underwent surgery for adhesiolysis at an average cost of $65,955 each, for a total of over $25 billion. Of these, 42,126 patients were readmitted to the hospital within 30 days of surgery, an 11% rate for hospital readmission. Additionally, 100,335 subjects had surgical small bowel resections and 15,050 subjects were readmitted within 30 days of the bowel resection, a 15% readmission rate. Bowel resection surgery patients averaged 14.2 days in the hospital in 2010, at an average cost of $114,175 [5], a total of $11.5 billion. A large previously published clinical study followed adult patients 10 years after surgery and found that more than one-third of the surgical small bowel resection patients underwent additional surgery due to adhesions within the study time frame [6]. Thus, adhesion related disease causes significant surgical efforts and hospital resources and comprises major expenditures each year. There is often considerable pain and negative impact on the patients' quality of life from these recurrent obstructions and hospital readmissions.

In the absence of bowel strangulation and peritonitis, the current recommendations to manage adhesive small bowel obstructions apply diagnostic imaging, clinical symptomology, and supportive care for the first 72 hours to allow the blockage adequate time to resolve independent of surgery [4]. While effective in treating the current obstruction, this approach does not address the internal adhesions or the risk of subsequent bowel obstructions. The only treatment currently available to treat adhesive bowel obstructions is surgery, which initiates the formation of new adhesions. A number of surgical techniques, modifications, and adhesion preventing medications and barriers have been investigated but, to date, no solution has been shown to completely prevent the formation of adhesions [7–13]. Thus, any conservative therapy that reduces adhesions or decreases the risk of bowel obstruction in the absence of surgery is of significant importance.

Manual physical therapy (mPT) is used to treat adults with a wide variety of adhesive conditions including burns, adhesive capsulitis, radiculopathy, pain, infertility, and lessening of scars [14–24] and has shown promise in preventing adhesion formation in animal models [25, 26]. The Clear Passage Approach (CPA), a mPT protocol hypothesized to deform the adhesions that cause SBO episodes, has been demonstrated previously in case reports to negate the need for surgery in patients with recurrent SBOs and other adhesion related diseases [27–29].

Observational care and bowel rest are preferred in nonemergency cases of SBO, supporting the desire to use the least invasive method to treat each subject. Therefore, a noninvasive technique to treat symptoms related to SBOs has precedent. In this study, we report on the use of the CPA, a manual physical therapy protocol, to treat abdominal and pelvic adhesions causing SBOs and improve the quality of life (QOL) of study subjects.

## 2. Patients and Methods

### 2.1. Subject Eligibility Criteria

Subjects with a history of adhesive bowel obstruction were included in this study. Subjects were screened following the standard clinic contraindications for CPA treatment including BMI over 36, active infection, abnormal ovarian cysts, surgery within the last 90 days, and bleeding disorders. Subjects with active or end stage cancer were excluded from this study. Each subject was provided with a written informed consent as is standard for the clinical practice. This study was conducted in accordance with the Declaration of Helsinki and the protocol was approved by the MaGil Institutional Review Board (SBO-2013-VAL). A total of 27 subjects treated between the years of 2011 and 2012 were eligible and included in the study. One female subject was lost to follow-up and is not included in the analysis.

### 2.2. Study Design

This was a single center prospective study designed to assess the changes in quality of life after CPA treatment, with all treatment occurring in a private physical therapy clinic located in Gainesville, Florida. Each subject served as their own control for the purpose of evaluating posttreatment change; all subjects received the CPA treatment. Subjects provided previous medical history and records; no diagnoses or radiological evaluations were performed as a part of the study. The QOL measures were performed in an observational manner using the validated paper based SBO Questionnaire (SBO-Q) [30]. All six domains of the SBO-Q were analyzed in this study (diet, pain, gastrointestinal (GI) symptoms, medication, QOL, and pain severity). Questionnaires were completed prior to treatment for the control measure. The posttreatment questionnaires were sent to the subjects 90 days after treatment, with the average day of completion at 117.4 ± 25.9 days after treatment.

Data from historical normal individuals collected during the validation study of the questionnaire was used as the range for “normal” in this study as previously described [30]. Pain numerical scores using the FDA standard 11-point scale were considered different only when a change of 2 points or greater was observed from pre- to posttreatment questionnaires [31].

### 2.3. Sample Size Calculation

For computing the necessary sample size, we made some simplifying assumptions. We assumed differences in the 6-domain scores before and after treatment are independent and follow a normal distribution. For all but one of the SBO-Q domains we assume that the mean difference is zero (i.e., no effect of the treatment), but for one domain we assume that the treatment lowers the domain score by an average of 3 points (i.e., for that one domain, the treatment delivers a significant improvement by 3 points) and we assumed that the standard deviation of each difference is 4 points. We then simulated the power by looking at the proportion of time that the multiplicity adjusted (for the 6 domain comparisons) one-sided *p* value for the one domain where we assumed a treatment effect is less than 2.5%. When selecting a sample size of 25, we reach a power of detecting a significant difference in at least one domain of 81%. A total of 27 subjects were included to allow for a 10% loss to follow-up.

### 2.4. Treatment

The manual physical therapy protocol utilized in this study was the CPA, an intensive treatment protocol that uses techniques from a variety of manual physical therapy modalities to treat the subject in an individualized, whole body approach. Subjects received 4 hours of manual physical therapy each day, with the typical subject completing 20 hours of treatment over the course of 5 days.

The CPA is comprised of over 200 individual techniques that focus on deforming or detaching adhesions to increase the mobility of adhered tissues and organs. The therapy accomplishes this by the use of various site-specific pressures across restrictive bands of adhered tissues and structures, working progressively deeper. Mobility was restored at the most superficial tissues using myofascial release [32]. Adhesions within and between organs and in interstitial spaces within the viscera were addressed using the Wurn Technique [29]. Decreased organ motility was addressed using visceral manipulation [32]. The amount of force and time the force was applied to each area had the potential to be significant but was maintained within the tolerance of the subject. In accordance with guidelines of the American Physical Therapy Association, detailed clinical treatment records were maintained throughout the course of therapy.

### 2.5. Subject Monitoring

Subjects treated with the CPA were monitored daily for changes in pain, diet, bowel habits, and overall well-being as is standard practice. Adverse events were monitored by the treating physical therapists during the course of treatment. There were no adverse events reported over the course of this study.

### 2.6. Statistical Tests

We used permutation tests throughout to test global (overall) and individual hypotheses, selecting the minimum *p* value based on a paired *t*-statistic as a test statistic [33]. A permutation test does not make any assumptions about the distribution of the response scores or their differences in the pre- and posttherapy measurements, such as assuming normality. If the null hypothesis of no therapy effect was true, then the differences in pre- and posttherapy measurements would be randomly distributed around 0.

When rejecting a global (overall) hypothesis of no effect, we are naturally interested in determining the factors that contribute to the significant overall improvement. In this paper, we present multiplicity adjusted *p* values for the individual hypothesis. The multiplicity adjustment is necessary to account for the fact that several hypotheses are tested simultaneously and to control the overall type I error of at least one false statement at the desired 2.5% level, with *p* ≤ 0.025 being significant.

The statistical package R (R Foundation for Statistical Computing, Vienna, Austria) was used for all statistical analyses.

## 3. Results

### 3.1. Subjects

A total of 27 subjects were enrolled in this study. Demographics for study subject are located in Table 1. There was one loss to follow-up due to the subject not completing the follow-up SBO-Q that was not included in the analysis. Due to a scheduling issue, one subject did not undergo a discharge evaluation and therefore is not included in the range of motion analysis. All subjects with follow-up completed the SBO-Q.

### 3.2. SBO Questionnaire Analysis

A statistical analysis for the data gathered from the pre- and posttreatment SBO-Q was conducted. In a first step, we investigated if there was an overall treatment effect, trying to answer the question whether CPA therapy resulted in a significant positive improvement in at least one of the response scores as measured by the SBO-Q. In particular, we tested the null hypothesis that none of the subject's responses to the survey questions were significantly lower after therapy as compared to before therapy. The alternative hypothesis was that there was a significant change in the positive direction (i.e., a tendency for lower scores on the survey) for at least one of the questions. The data on the survey scores for the 26 subjects before and after treatment provide sufficient evidence (one-sided permutation *p* value < 0.001) to reject the null hypothesis and conclude that there was a significant therapeutic effect. This means that for at least one of the survey questions, the mean posttherapy score was significantly lower than the mean pretherapy score, indicating an improvement. Missing data from any question/subject was removed from the overall analysis.

### 3.3. Quality of Life

To investigate which domain(s) may be responsible for the overall improvement, we conducted a domain analysis. Grouped by survey questions, the domains refer to the six general categories shown in Table 2. The pain, quality of life, and pain severity domains all demonstrated significant improvement of average scores after treatment with *p* ≤ 0.001. We also saw a marginally significant effect in the GI symptom domain (*p* = 0.0258), indicating that there were suggestive improvements also for this domain. No significant improvements were seen for the diet or medication domain.

In a final step, we investigated which of the 37 questions on the survey showed a significant improvement in the mean score after therapy; see Table 2. Results indicated a significant improvement in the mean scores on many of the quality of life questions. For instance, after therapy, subjects missed significantly fewer days of work due to their condition (*p* = 0.0018), were more able to have a normal social life (*p* = 0.0095), and participated more in normal daily activities (*p* = 0.0023). They also worried significantly less about having another bowel obstruction after therapy (*p* = 0.0064). There was also some indication that their sex life improved after therapy (*p* = 0.0662). All these factors contribute to the significant domain effect for the quality of life domain we observed in the previous paragraph.

Similarly, the significant pain domain effect is mostly due to subjects showing significantly fewer days of experiencing general pain (*p* = 0.0216), head or neck pain (*p* = 0.0100), and pain after eating (*p* = 0.0413). The significant domain effect for pain severity was mostly driven by a significant reduction in the duration of the maximum pain (*p* = 0.0006) 90 days after therapy as compared to before treatment and a significant lower average pain after treatment (*p* = 0.0599).

We could not observe a statistically significant reduction in many of the questions in the “GI symptom” domain, but we did find that, on average, subjects reported fewer days where eating or drinking caused them to swell, bloat, or have gas after their treatment (*p* = 0.0388). We also did not see a significant improvement in the number of days subjects had to take medication or had problems with their diets. This is not surprising given the fact that subjects who entered the study took medication or had issues with their diets only a few days per month, so generally only small improvements were observed.

The distribution of degree of impact on QOL for each of the domains was assessed for observation of distribution of subjects after treatment.  Table 3 shows the total number of subjects in each domain by the SBO-Q established quartiles for classification of degree of impact on life, allowing for observation of clinically relevant trends. There was a clear trend of improvement in all domains, with more subjects classified as “normal” after treatment, with only 1 measure having any subject classified as “severely impacted” in one domain after CPA treatment, compared to 18 separate measures classified as “severely impacted” before CPA treatment.

### 3.4. Range of Motion

All subjects underwent a comprehensive physical therapy initial evaluation that included measurements for range of motion (ROM). Based upon observations in the SBO patient population by this group, subjects with a history of SBO typically have decreases in ROM associated with mechanical factors such as abdominal and/or pelvic adhesions; therefore, these measurements are integrated in the assessment of subject outcome after CPA treatment. Included in these measurements are flexion of the trunk, extension of the trunk, left and right side bending, and rotation to both left and right sides [34–37].

All subjects with decreased ROM demonstrated improvement for at least one of the measurements presented in Table 4. Furthermore, 28.6% of total measurements exhibiting a decreased ROM returned to normal. Although not all subjects had a normal ROM after treatment, every subject demonstrated overall improvement.

Statistical analysis of changes showed a significant increase in the mean ROM for at least one of the six measurements in Table 4 (*p* < 0.001). After adjusting for multiplicities, we found a significant increase in the mean ROM for each of the six measures.

The improvement in extension suggests that the adhesions present in the abdomen and pelvis that prevented the subject from bending backwards improved for trunk extension following treatment. The improvements in side bending and rotation also suggest that the decreased adhesions present in the abdomen and pelvis had a decreased effect on trunk ROM. These findings indicate that decreased trunk rotation may be used as a noninvasive indicator of abdominal adhesions and that improvements in trunk ROM measurements may be an appropriate predictor for degree of adhesion deformation post CPA treatment.

## 4. Conclusions

It is widely accepted that the adhesions that cause SBO and symptomology in subjects are typically caused from prior abdominal or pelvic surgery [4, 38]. The current study is the first prospective study to assess the changes in quality of life for subjects with a history of SBO treated with the CPA using the validated SBO-Q [30]. It introduces the option of using a nonsurgical approach to treat the adhesions that cause SBO in stable, nonemergency situations.

This manual physical therapy regimen presents few risks as compared to surgical approaches and research thus far supports the observation that, unlike surgical approaches, adhesions are not reformed after treatment with the CPA [22]. The results from this study suggest that the CPA can be used to treat adhesions and scar adherence safely in the recurrent SBO subject population, demonstrating significant improvements in overall pain, quality of life, and severity of pain and improvements in gastrointestinal symptoms being suggestive. Further, patients who were previously concerned about having another SBO episode reported a significant decrease in that concern three months after treatment. The improvement in quality of life for this patient population is positive as a decline in quality of life is the largest complaint from patients with bowel obstructions.

Although improvements in diet and requirements for medication were not significant, trends demonstrated improvement for all subjects with follow-up in this study. It is expected that in a larger study with more subjects there will be improvements in diet and medication requirements given the general trends of improvements in those subjects classified as having an impact before treatment (Table 3).

Based upon changes in range of motion, it is inferred that tissue and organ mobility were improved as the subjects demonstrated an increased range of motion in active movement tests. This inference is based upon the ability of the patient to move with decreased pain and tissue rigidity improvements, similar to that of joint mobility assessment. Improvements in range of motion allowed subjects to perform daily tasks more easily and contributed positively to their overall QOL.

This study also supports the use of the CPA in the pediatric SBO subject population. Literature for pediatric subjects with SBO following abdominal surgery is not readily available compared to studies following adult populations. However, several retrospective reviews of pediatric surgical cases for SBO report varying rates of adhesions causing SBO following prior surgeries [1, 39, 40]. There is no lifelong cure currently available; thus these patients must deal with side effects and manage symptoms for the rest of their lives, with the additional concern of growth and physical development impacts from restrictive scar tissue. The positive results from the two pediatric subjects included in this study support the need for additional studies for treatment of pediatric SBO subjects with CPA treatment.

The limitations of this study include the small number of subjects; a larger study will likely offer more power. Due to the lack of available nonsurgical diagnostics to assess adhesion presence in the body, the improvement in self-reported symptoms and improvements in ROM were used as the outcome measures in this study. Generalized symptom improvement is limited to this population subset with a history of nonemergent adhesive SBO.

## Figures and Tables

**Table 1:** Study participant characteristics.

Characteristics	Number of subjects
Age, years	
Median = 53	
Range = 10.7–89.4	
Sex	
Male	10
Female	17
Race	
Caucasian	26
Black	0
Hispanic	1
Native American	0
Other	0
Marital status	
Married	15
Single	8
Widowed	1
Divorced	3
Number of previous surgeries	
0	3
1-2	9
3–5	7
>6	7
Number of prior partial bowel obstructions	
0	3
1–10	16
11–20	5
>20	5
Number of prior total bowel obstructions	
0	13
1	5
2	3
3	3
4	2
Number of hours of treatment	
Median = 20	
Range = 12–40	

**Table 2:** Average scores and permutation *p* values for each domain and question in the SBO-Q to assess changes in scores before and after CPA treatment.

Domain Question abbreviation (survey question)	Before treatment score mean (s.d.)	After treatment score mean (s.d.)	Score difference mean (s.d.)	Number of subjects	*p* value^*∗*^
*Diet*					**0.8191**
Liquid (I was on a totally liquid diet)	3.46 (0.90)	3.81 (0.40)	−0.35 (0.80)	26	1.0000
Soft (I was on a soft food diet)	2.65 (1.32)	3.42 (1.17)	−0.77 (1.53)	26	1.0000
Solid (I could easily ingest and digest solid food)	2.48 (1.39)	2.38 (1.81)	0.16 (1.68)	25	0.9031
Anything (I could eat whatever I liked, without problems)	0.92 (1.52)	1.85 (1.64)	−0.92 (1.830)	26	1.0000

*Pain*					**0.0087**
General (I had pain)	2.04 (1.73)	1.12 (1.37)	0.92 (1.47)	26	0.0216
Upper GI (I had pain at or above my belly button)	1.50 (1.56)	0.69 (1.05)	0.81 (1.50)	26	0.0662
Lower GI (I had pain below my belly button)	1.31 (1.52)	0.85 (1.22)	0.46 (1.24)	26	0.3930
BM (I had pain with bowel movements)	0.81 (1.06)	0.50 (1.14)	0.31 (1.16)	26	0.6378
Head_Neck (I experienced head or neck pain)	1.08 (1.16)	0.50 (1.10)	0.58 (0.86)	26	0.0100
Migraine (I had migraine headache(s))	0.15 (0.46)	0.19 (0.57)	−0.04 (0.53)	26	0.9814
Coccyx (I experienced tailbone (coccyx) pain)	0.54 (1.07)	0.04 (0.20)	0.50 (1.10)	26	0.1605
Eating (Eating caused my abdomen to hurt)	1.58 (1.50)	0.77 (1.14)	0.81 (1.41)	26	0.0413
Drinking (Drinking liquids caused my abdomen to hurt)	0.58 (1.14)	0.31 (0.97)	0.27 (0.96)	26	0.6040
Back (I had back pain)	1.69 (1.62)	1.15 (1.41)	0.54 (1.10)	26	0.1263

*GI symptoms*					**0.0258**
Nausea (I had nausea after eating)	0.80 (1.04)	0.31 (0.62)	0.56 (1.04)	25	0.0850
Vomit (I vomited after eating)	0.35 (0.85)	0.04 (0.20)	0.31 (0.84)	26	0.4006
GI_spasm (I experienced digestive spasm)	0.92 (1.35)	0.42 (0.90)	0.50 (1.48)	26	0.4650
Constipation (I had constipation)	1.38 (1.30)	0.85 (1.08)	0.54 (1.58)	26	0.4623
Diarrhea (I had diarrhea)	1.04 (1.18)	0.85 (0.97)	0.19 (0.94)	26	0.7867
BS_JLM (I eliminated blood-stained, or jelly-like mucus)	0.20 (0.58)	0.27 (0.67)	−0.08 (0.64)	25	0.9814
Gas_Bloat_Dist (I had gas/bloating/distension)	2.04 (1.43)	1.28 (1.28)	0.80 (1.38)	25	0.0497
Inc_Sounds (I had increased bowel sounds)	1.69 (1.46)	1.00 (1.17)	0.69 (1.57)	26	0.1802
No_BM (I was unable to have bowel movements when I wanted or needed to go)	1.31 (1.41)	0.77 (1.27)	0.54 (1.14)	26	0.1321
Ab_BM (My bowel movements looked abnormal)	1.58 (1.68)	0.96 (1.22)	0.62 (1.44)	26	0.2276
Eat_Bloat (Eating or drinking caused me to swell, bloat, or have gas)	1.96 (1.48)	1.24 (1.36)	0.72 (1.21)	25	0.0388

*Medication*					**0.8191**
Meds (I took medications for my symptoms)	1.81 (1.88)	1.77 (1.75)	0.04 (2.03)	26	0.9689

*Quality of life*					**0.0016**
Off_Work (I was unable to work due to my condition)	1.28 (1.37)	0.29 (0.62)	1.04 (1.27)	24	0.0018
Off_Social (I was unable to have a normal social life due to my condition)	1.35 (1.57)	0.42 (0.99)	0.92 (1.35)	26	0.0095
Off_Sex (My sex life suffered due to my condition)	1.68 (1.76)	0.57 (1.16)	0.84 (1.30)	19	0.0662
Off_Daily_Function (I had decreased ability to participate in normal daily activities due to my condition)	1.65 (1.52)	0.54 (1.14)	1.12 (1.45)	26	0.0023
Off_Eat_Out (I would be reluctant to eat at a restaurant or a friend's house due to my condition)	1.62 (1.55)	0.85 (1.46)	0.77 (1.53)	26	0.1072
Massage_worry (I felt that I could decrease my own symptoms using my hands)	3.31 (1.05)	3.08 (1.24)	0.17 (1.20)	24	0.8785
Worry (I worried about having another bowel obstruction)	2.23 (1.63)	1.12 (1.28)	1.12 (1.56)	26	0.0064

*Pain severity*					**0.0006**
Duration_Pain (The duration of the worst pain)	4.20 (1.44)	2.50 (2.14)	1.76 (2.05)	25	0.0006
Recent_Max_Pain (Maximum pain in bowels over the last 4 weeks)	6.10 (3.37)	4.15 (2.96)	1.82 (4.19)	25	0.2276
Recent_Min_Pain (Minimum pain in bowels over the last 4 weeks)	2.12 (2.24)	1.42 (1.36)	0.68 (2.54)	25	0.6378
Recent_Avg_Pain (Average pain in bowels over the last 4 weeks)	3.96 (2.76)	2.08 (1.90)	1.84 (3.24)	25	0.0599

^*∗*^One-sided multiplicity adjusted.

s.d. = standard deviation.

**Table 3:** Distribution of subjects classified by cumulative scores for each domain per the standard scoring of the SBO-Q. There was a clear shift in the number of subjects to the classification of normal after treatment.

Domain	Before treatment				After treatment			
	Normal	Slight impact	Moderate impact	Severe impact	Normal	Slight impact	Moderate impact	Severe impact
Diet	6	9	6	5	13	4	9	0
Pain	14	6	5	1	22	2	1	1
GI symptoms	12	10	4	0	19	4	3	0
QOL	8	7	5	6	20	2	4	0
Medication	14	3	3	6	24	1	1	0

**Table 4:** Analysis for range of motion measurements before and after CPA treatment.

	Range of motion measure (normal°)	Pretreatment (*N* = 25) mean (s.d.)	Posttreatment (*N* = 25) mean (s.d.)	*p* value^*∗*^
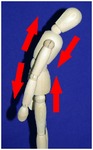	Flexion (80°)	69.20 (10.07)	73.80 (6.17)	0.000003
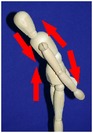	Extension (25°)	14.68 (7.72)	20.60 (5.27)	0.000008
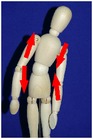	Left side bending (45°)	35.20 (8.95)	40.40 (7.63)	0.000005
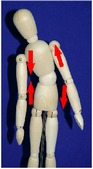	Right side bending (45°)	35.20 (9.63)	40.60 (6.82)	0.00003
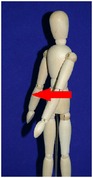	Left rotation (45°)	32.80 (10.81)	39.40 (7.54)	0.000008
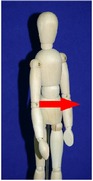	Right rotation (45°)	34.40 (10.54)	39.00 (7.50)	0.00003

^*∗*^One-sided multiplicity adjusted.

s.d. = standard deviation.
